# Supplementary material for: Pathway choice in the alternative telomere lengthening in neoplasia is dictated by replication fork processing mediated by EXD2’s nuclease activity
Source: Nat Commun. 2023 Apr 27;14:2428. doi: 10.1038/s41467-023-38029-z (PMC10140042; doi:10.1038/s41467-023-38029-z)
Supplement: Supplementary file 3 — Reporting Summary [file 41467_2023_38029_MOESM3_ESM.pdf]

nature portfolio

Corresponding author(s): Prof W. Niedzwiedz, Prof S Gagos

Last updated by author(s): Mar 21, 2023

Reporting Summary

Nature Portfolio wishes to improve the reproducibility of the work that we publish. This form provides structure for consistency and transparency in reporting. For further information on Nature Portfolio policies, see our Editorial Policies and the Editorial Policy Checklist.

Statistics

For all statistical analyses, confirm that the following items are present in the figure legend, table legend, main text, or Methods section.

n/a	Confirmed	
☐	☒	The exact sample size (*n*) for each experimental group/condition, given as a discrete number and unit of measurement
☐	☒	A statement on whether measurements were taken from distinct samples or whether the same sample was measured repeatedly
☐	☒	The statistical test(s) used AND whether they are one- or two-sided<br>*Only common tests should be described solely by name; describe more complex techniques in the Methods section.*
☒	☐	A description of all covariates tested
☒	☐	A description of any assumptions or corrections, such as tests of normality and adjustment for multiple comparisons
☐	☒	A full description of the statistical parameters including central tendency (e.g. means) or other basic estimates (e.g. regression coefficient) AND variation (e.g. standard deviation) or associated estimates of uncertainty (e.g. confidence intervals)
☐	☒	For null hypothesis testing, the test statistic (e.g. *F*, *t*, *r*) with confidence intervals, effect sizes, degrees of freedom and *P* value noted<br>*Give P values as exact values whenever suitable.*
☒	☐	For Bayesian analysis, information on the choice of priors and Markov chain Monte Carlo settings
☒	☐	For hierarchical and complex designs, identification of the appropriate level for tests and full reporting of outcomes
☒	☐	Estimates of effect sizes (e.g. Cohen's *d*, Pearson's *r*), indicating how they were calculated

Our web collection on statistics for biologists contains articles on many of the points above.

Software and code

Policy information about availability of computer code

Data collection: Images were acquired with a 3i Advance spinning disc microscope using dedicated software or a Zeiss Axio-Imager Z1 (Zeiss) or Zeiss LSM510 microscope using dedicated software or an Oxford Optronix Gelcount machine and Gelcount software. FACS data was acquired using a BD LSR II flow cytometer and FACSDiva software (Becton-Dickinson).

Data analysis: Image analysis was carried out in FIJI (ImageJ, version 2.1.0/1.53c) or Metasystems Isis analysis software V.5. Statistical analysis was carried out using Graphpad PRISM 9.5.1 software.

For manuscripts utilizing custom algorithms or software that are central to the research but not yet described in published literature, software must be made available to editors and reviewers. We strongly encourage code deposition in a community repository (e.g. GitHub). See the Nature Portfolio guidelines for submitting code & software for further information.

Data

Policy information about availability of data

All manuscripts must include a data availability statement. This statement should provide the following information, where applicable:

- Accession codes, unique identifiers, or web links for publicly available datasets
- A description of any restrictions on data availability
- For clinical datasets or third party data, please ensure that the statement adheres to our policy

Raw images have been uploaded to mendeley data DOI:10.17632/dmk886xwxw.1

Field-specific reporting

Please select the one below that is the best fit for your research. If you are not sure, read the appropriate sections before making your selection.

☒ Life sciences ☐ Behavioural & social sciences ☐ Ecological, evolutionary & environmental sciences

For a reference copy of the document with all sections, see nature.com/documents/nr-reporting-summary-flat.pdf

Life sciences study design

All studies must disclose on these points even when the disclosure is negative.

Sample size	All data described were generated from multiple biological replicates with sample sizes of sufficient size to be applicable for student's t-test and Mann-Whitney analysis.
Data exclusions	No data were excluded.
Replication	The majority of experiments were performed at least in triplicate. In some instances, as indicated in the text, experiments were performed in duplicate. In all cases repeated experiments were successful.
Randomization	Samples were randomly assigned to different groups (e.g. control vs. treated).
Blinding	Blinding was applied for scoring of microscopy images, where appropriate. For other analyses such as western blotting, colony formation assays there was no need for blinding as these techniques either did not require blinding or operate via automated analysis of images acquired.

Reporting for specific materials, systems and methods

We require information from authors about some types of materials, experimental systems and methods used in many studies. Here, indicate whether each material, system or method listed is relevant to your study. If you are not sure if a list item applies to your research, read the appropriate section before selecting a response.

Materials & experimental systems

n/a	Involved in the study	
☐	☒	Antibodies
☐	☒	Eukaryotic cell lines
☒	☐	Palaeontology and archaeology
☒	☐	Animals and other organisms
☒	☐	Human research participants
☒	☐	Clinical data
☒	☐	Dual use research of concern

Methods

n/a	Involved in the study	
☒	☐	ChIP-seq
☒	☐	Flow cytometry
☒	☐	MRI-based neuroimaging

Antibodies

Antibodies used

Primary Antibodies for western blotting were as follows:
α-Tubulin (Sigma, B-5-1-2; T5168, 1:100,000)
BLM (Bethyl, A300-110A 1:2000)
DNA2 (Abcam ab962488, 1:1000)
EXD2 (Sigma, HPA005848, 1:1000)
MCM2 (Abcam, ab4461, 1:10,000)
MRE11 (Abcam, ab214, 1:1000)
MUS81 (Abcam, ab14387 1:1000)
POLD3 (Abnova, H00010714-M01, 1:500)
RAD52 (28045-1-AP, Proteintech, 1:2000)
SMARCAL1 (Santa Cruz, sc-376377 1:1000)
SLX4 (University of Dundee, DU16029, 1:200)
Vinculin (Thermo-Fisher, MA5-11690, 1:1000).
Secondary Antibodies for western blotting were as follows:
Goat Anti-Mouse Immunoglobulins/HRP (affinity isolated) (P0447, Dako, 1:2000)
Goat Anti-Rabbit Immunoglobulins/HRP (affinity isolated) (P0448, Dako, 1:5000)
Rabbit anti-Sheep IgG (H+L) Secondary Antibody, HRP (ab6747, Abcam, 1:500)

Primary antibodies employed for immunofluorescence were as follows:
gammaH2AX (JBW301, Millipore, 1:500),

53BP1 (MAB3802, Millipore, 1:1000)
MRE11 (ab214, Abcam, 1:500)
MUS81 (sc53382, Santa Cruz Biotechnology, 1:200)
PML (E-11 sc-377390, Santa Cruz Biotechnology, 1:500)
PML (Ab96051, Abcam, 1:500)
POLD3 (Abnova, H00010714-M01, 1:500)
RAD51 (Bioacademia, 70-002; 1:500)
RAD52 (Sheep, a kind gift from Prof T. Halazonetis University of Geneva, Switzerland, 1:100)
SLX4 (University of Dundee, DU16029, 1:200)
SMARCAL1 (Santa Cruz, sc-376377 1:500)
TRF1 (Rabbit, #6839 a gift from Prof J. Karlseder-The Salk Institute for Biological Studies. La Hoya, USA)
TRF2 (A300-796A-T, Thermo-Fisher, 1:500, PA1-41023. Thermo-Fisher 1:500)
TRF2 Rabbit #6841 1:500, from Prof J. Karlseder)
RPA1 (Ab-3; Calbiochem 1:1000)

Secondary antibodies employed for immunofluorescence were as follows

Donkey anti-Rabbit IgG (H+L) Highly Cross-Absorbed Secondary Antibody, Alexa Fluor™ 647 (A-31573, Thermo-Fisher, 1:400).
Donkey anti-Rabbit IgG (H+L) Highly Cross-Adsorbed Secondary Antibody, Alexa Fluor™ 555
(A-31572, Thermo-Fisher, 1:400).
Donkey anti-Rabbit IgG (H+L) Highly Cross-Adsorbed Secondary Antibody, Alexa Fluor™ 488 (A-21206, Thermo-Fisher, 1:200).
Donkey anti-Mouse IgG (H+L) Highly Cross-Adsorbed Secondary Antibody, Alexa Fluor™ 488 (A-21202, Thermo-Fisher, 1:200).
Donkey anti-Mouse IgG (H+L) Highly Cross-Adsorbed Secondary Antibody, Alexa Fluor™ 555
(A-31570, Thermo-Fisher, 1:400).
Goat anti-Rabbit IgG (H+L) Cross-Adsorbed Secondary Antibody, Alexa Fluor™ 568 (A-11011), Thermo-Fisher, 1:400).

Primary antibodies used for Proximity Ligation assay were as follows:

53BP1 (MAB3802, Millipore, 1:1000)
FLAG (Sigma M2, 1:500)
GFP (Roche 11 814 460 001, 1:500)
TRF1 (Rabbit, #6839 from Prof J. Karlseder 1:200)
TRF2 (Rabbit #6841 from Prof J. Karlseder, 1:500)
TRF2 (A300-796A-T 1:200)

Validation

All commercial antibodies have been validated by their manufcaturers.

TRF1 (Rabbit, #6839) and TRF2 (Rabbit #6841) from Prof J. Karlseder have been validated and published in the literature1
RAD52 antibody from Prof T. Halazonetis has been validated and published in the literature2.

1 Hayashi, M. T., Cesare, A. J., Rivera, T. & Karlseder, J. Cell death during crisis is mediated by mitotic telomere deprotection. Nature 522, 492-496 (2015). https://doi.org:10.1038/nature14513
2 Sotiriou, S. K. et al. Mammalian RAD52 Functions in Break-Induced Replication Repair of Collapsed DNA Replication Forks. Molecular cell 64, 1127-1134 (2016). https://doi.org:10.1016/j.molcel.2016.10.038

Eukaryotic cell lines

Policy information about cell lines

Cell line source(s)

HeLa and U2OS cells obtained from Dr F. Esashi (University of Oxford, UK) and were originally purchased from ATCC (HeLa CCL-2 and U2OS HTB-96, respectively).

U2OS stably expressing GFP were obtained from Prof. S. Jackson (University of Cambridge).

The SV-40 large T-antigen transformed ALT cell lines GM-847, VA-13, and IMR-90 were donated by Dr A. Londoño-Vallejo (Institute Curie, Paris, France).

The VA-13-h-Tel cell line that stably expresses human telomerase RNA component (hTERC) and human telomerase reverse transcriptase (hTERT) with reconstituted telomerase activity1 was a gift from Prof J. W. Shay (UT Southwestern Medical Center, Dallas, TX, USA).

The ALT+ liposarcoma Lisa-2 cells were kindly provided by Dr D. Broccoli (Fox Chase Cancer Center, Philadelphia, PA, USA).

The U2OS EJ2-GFP cells were a kind gift of Prof J. Stark (City of Hope, Department of Cancer Genetics and Epigenetics, Duarte, CA, USA)2.

EXD2-/- U2OS cells were generated as described previously3.

U2OS cells overexpressing cyclin E in a tetracycline-dependent manner and RAD52-/- cells generated by CRISPR-Cas9 in this background, were a kind gift from Prof T. Halazonetis (University of Geneva, Switzerland)4.
U2OS cells stably expressing GFP-EXD2, FLAG-HA WT or nuclease-dead EXD2 were generated previously by the Niedzwiedz lab3,5.

Pools of EXD2-/- U2OS cells stably expressing FLAG- HA EXD2 WT or nuclease dead protein were generated by transfection with plasmid constructs encoding WT or nuclease dead EXD2 in the pHAGE-N-Flag–HA vector backbone and selection with puromycin (1µg/ml) in this study.

1 Ford, L. P. et al. Telomerase can inhibit the recombination-based pathway of telomere maintenance in human cells. The Journal of biological chemistry 276, 32198-32203 (2001). https://doi.org:10.1074/jbc.M104469200
2 Gunn, A. & Stark, J. M. I-SceI-based assays to examine distinct repair outcomes of mammalian chromosomal double strand breaks. Methods Mol Biol 920, 379-391 (2012). https://doi.org:10.1007/978-1-61779-998-3_27
3 Nieminuszczy, J. et al. EXD2 Protects Stressed Replication Forks and Is Required for Cell Viability in the Absence of BRCA1/2. Molecular cell (2019). https://doi.org:10.1016/j.molcel.2019.05.026
4 Sotiriou, S. K. et al. Mammalian RAD52 Functions in Break-Induced Replication Repair of Collapsed DNA Replication Forks. Molecular cell 64, 1127-1134 (2016). https://doi.org:10.1016/j.molcel.2016.10.038
5 Broderick, R. et al. EXD2 promotes homologous recombination by facilitating DNA end resection. Nature cell biology 18, 271-280 (2016). https://doi.org:10.1038/ncb3303

Authentication

HeLa and U2OS cells were obtained from and authenticated by ATCC. U2OS cells ectopically expressing tagged proteins or rendered knockout for EXD2 were derived from these.
U2OS EJ2-GFP cells were provided by the Stark lab and have been validated as described in the literature2.
U2OS cells overexpressing cyclin E in a tetracycline-dependent manner and RAD52-/- cells generated by CRISPR-Cas9 in this background were validated by Prof T. Halazonetis group and are published in the literature4.

The SV-40 large T-antigen transformed ALT cell lines GM-847, VA-13, VA-13+hTel and IMR-90 were authenticated by karyotyping in the Gagos lab by an American Board of Medical Genetics certified Clinical Cytogeneticist.

Mycoplasma contamination

All cell lines used tested negative for Mycoplasma contamination.

Commonly misidentified lines
(See ICLAC register)

None of the cell lines used are listed in the database of commonly misidentified cell lines.
